# A narrative review of exercise intervention mechanisms for post-traumatic stress disorder in veterans

**DOI:** 10.3389/fpubh.2024.1483077

**Published:** 2025-01-07

**Authors:** Fang Zhao, Chuchen Liu, Zhiyi Lin

**Affiliations:** ^1^School of Physical Education & Health, Nanning Normal University, Nanning, China; ^2^School of Physical Education & Sport Science, Fujian Normal University, Fuzhou, China

**Keywords:** exercise intervention, PTSD, veterans, exercise prescription, mental health

## Abstract

Post-traumatic stress disorder (PTSD) severely disrupts the daily lives of veterans and active duty personnel and may influence their suicidal behaviour. This study provides insight into existing research on PTSD in veterans through a narrative review. Exercise was found to reduce PTSD symptoms in veterans at both psychological and physiological levels, which in turn inhibits their suicidal tendencies. At the psychological level, exercise improved veterans’ Subjective Well-Being and Psychological Well-Being, and at the physiological level, it improved veterans’ brain structure, neuroendocrine system, and immune system. By combing these mechanisms in detail, we hope to provide theoretical support for the implementation of exercise interventions in the treatment of veterans with PTSD. However, it is important to note that the specifics of the exercise program, such as the optimal type, dosage, and duration to alleviate PTSD symptoms, remain unclear and require further research and exploration.

## 1 Introduction

Numerous medical reports have substantiated PTSD as a severe mental health issue, often entailing long-term and profound mental disorders alongside adverse behavioural responses like depression, anxiety, sleep disturbances, chronic pain, and even suicidality (1). Untreated, these conditions may progress into chronic, disabling diseases that impede normal post-military life (2). Notably, the engagement of military personnel in routine training and exercises could similarly contribute to PTSD development, potentially resulting in non-combat attrition. Consequently, addressing PTSD becomes a pivotal element in the comprehensive care of veterans and service members globally.

Presently, the world faces a heightened incidence of PTSD among military personnel, resulting in significant non-combat attrition due to ongoing political unrest and frequent conflicts. This condition has garnered attention from foreign governmental departments and numerous scholars owing to its high disability rates, frequent recurrence, and low cure rates among military personnel (3). There is evidence of a significant delay in seeking treatment for these military personnel with PTSD, for example, Murphy et al. (4) found that veterans may experience up to 12 years of mental anguish before seeking help. This is largely due to resistance to and barriers to PTSD treatment amongst military personnel: military personnel often view receiving psychiatric treatment as a sign of weakness (5) and they perceive it as a ‘stigma’ to be treated with psychological interventions (2), which stems from a high degree of masculinity, which stems from the residual and ongoing effects of a highly masculinized military environment. In this environment, soldiers are expected to be ‘tough, resilient and self-reliant’ and view ‘emotional weakness’ with suspicion, which may lead them to conceal the existence of mental health problems and avoid seeking help (6). These issues may lead military PTSD patients to reject traditional treatment programs or to “drop out” of traditional treatment programs. In summary, current evidence suggests that new approaches are needed to overcome barriers in the treatment of PTSD in veterans (7).

These studies have explored the role and necessity of exercise interventions in terms of their psychological mechanisms (e.g., subjective well-being versus psychological well-being) or physiological mechanisms (e.g., improvements in brain structure, neuroendocrine system and immune system) (8–10). However, relevant findings now lack a comprehensive study to unify and collate their content intervention mechanisms, to the detriment of future support for the treatment of PTSD symptoms in veterans. Against this backdrop, this paper aims to explore the primary symptoms among veteran patients, delineate the benefits of exercise interventions, and elucidate their underlying mechanisms of intervention. This article draws from a large compilation of research on exercise interventions for veteran patients. The purpose of the article is to provide theoretical support for the implementation of exercise interventions in the treatment of veteran patients with PTSD and to provide support for future exploration of the mechanisms of exercise interventions for PTSD.

## 2 Methods

In this review, we will focus on how exercise can effectively intervene in the treatment of PTSD in veterans, and as such, a narrative review approach has been adopted for our analyses. The purpose of this type of review is to provide a comprehensive and accessible overview of the progress of research on a particular issue (44, 45). Its narrative approach includes summarizing, comparing, explaining, and describing how sport can provide a positive impact on the treatment of PTSD in veterans.

Before proceeding with the literature review, it is necessary to clarify that the reference to ‘exercise intervention’ in this study refers to the instruction and modification of patients’ exercise processes through physical activity (11). The aim is to use physical activity up to a certain load to improve existing physical or mental fitness (11).

We started our literature search in the Web of Science database in March 2024. The keywords used for the search were ‘veterans’, ‘PTSD’ and ‘exercise intervention’, based on the primary focus of the study investigators and the research question. Article titles and abstracts were then used to screen for studies that were highly relevant to this research question. From relevant databases or publishers such as BSCO-ERIC, Scopus, MDPI, Taylor & Francis Online and Sage Journals (45), we conducted a secondary search for references mentioned in these articles based on careful reading. Until we were unable to sift through the literature from these studies that were helpful to us.

The review criteria were as follows (I) published in the Web of Science core repository; (II) focused on the performance of veterans with PTSD in sports; and (III) explicitly mentioned how they went through the sports intervention (46). After screening 193 papers with high relevance to this study, each author of this study identified the articles to be included in the review by reading them in detail.

After reading and organizing the content of the articles, all authors agreed to include the final 54 articles in this narrative review according to the following criteria: “whether it is an empirical study,” “whether it uses an exercise intervention,” “whether it elucidates the specific mechanism of the exercise intervention,” etc. The final 54 articles were screened. In addition to the articles that were agreed upon, 54 articles were agreed upon for inclusion in this narrative review through peer discussion and back-to-back analysis. Subsequently, we conducted a preliminary collation of the relevant content and found that this study consisted of the following two main categories:

(I) Exploring the effects of exercise interventions on the psychotherapy of PTSD in veterans, using the positive psychological effects of exercise interventions. These include adaptive and adventurous sports such as hiking and fishing, which alleviated their anxiety and depression, improved their concentration and pain tolerance, and had a positive effect on their SWB (12, 22). In addition, with the help of the novelty experience generated during the exercise, the establishment of new life roles for them, the removal of the negative labelling originally generated by PTSD, and the facilitation of social interaction, PWB was achieved, which contributed to the reduction and regression of their PTSD symptoms (13, 21).
(II) To explore the efficacy of exercise interventions in the physiological treatment of PTSD in veterans, based on the positive changes they produce in the body. Firstly, aerobic training has been shown to increase the volume of the hippocampus and the number of neurogenesis in the brain (14); secondly, exercise interventions can promote the release of cortisol and regulate the originally dysfunctional hypothalamic–pituitary axis (11); and finally, exercise interventions can reduce their levels of inflammatory activity by improving the regulation of hormones such as pro-inflammatory cytokines in the body (15).

After harmonizing the opinions of three authors, 54 articles were finally included in the study. The three authors then built on the literature study by further condensing the key words into one research question: Why and How Physical Activity Can Improve PTSD Symptoms in Veterans.

## 3 Mechanisms of exercise intervention for PTSD in military veterans

Exercise, being a prevalent physical activity in military training for service members, significantly reduces their anxiety regarding PTSD treatment. This heightened acceptance and adherence to exercise interventions among individuals in the military creates a favorable environment for implementing exercise interventions for veteran patients (3).

### 3.1 Psychological mechanisms of exercise interventions for PTSD in military veterans

Exercise offers primary psychological benefits in alleviating PTSD among military personnel by enhancing participants’ negative emotions, particularly anxiety and depression. Additionally, exercise contributes to ameliorating issues related to substance abuse, distraction, and social withdrawal resulting from PTSD (16). Building upon these findings, scholars have synthesized the psychological mechanism of exercise in PTSD improvement as a pathway for enhancing veterans’ Subjective Well-Being (SWB) and Psychological Well-Being (PWB) through the amelioration of PTSD symptoms achieved via exercise interventions (3).

#### 3.1.1 Exercise modulates SWB to improve PTSD symptoms

Caddick and Smith (3) defined SWB as an individual’s subjective satisfaction with life, encompassing the balance between positive and negative emotions experienced. They proposed that exercise influences SWB in military personnel through various mechanisms (3).

Existing research affirms the beneficial role of exercise in aiding injured or disabled veterans in managing psychological challenges stemming from post-war acquired disabilities. For instance, Burke et al. (22) scrutinized the psychosocial responses of four veteran patients who ascended Mount Kilimanjaro and found that their active cooperation and initiative during the climb constituted a positive coping strategy for PTSD, enhancing their SWB. It can be argued that the SWB of military personnel can be enhanced and their positive coping with PTSD symptoms can be facilitated through a six-phase adaptive training and self-challenging mountaineering activity over a period of time (9 days) (17). Additionally, Brittain and Green (18) observed that veterans’ participation in Paralympic training and competitions, because of the sense of honor of going to war for their country and their own sense of responsibility, helped them to come to terms with their physical disabilities and reduce the resulting psychological trauma.

Participation in exercise also engenders positive emotional experiences for veterans. For instance, Mowatt and Bennett (19) discovered that organizing ‘fly-fishing’ activities for military personnel reduced their PTSD symptoms related to ‘regret’ and ‘guilt.’ The sense of ‘guilt’ in their PTSD symptoms gave way to positive emotions like happiness, enjoyment, and relaxation, effectively mitigating the ‘depression’ and ‘anxiety’ that had been pervasive in their daily lives (19). Essentially, performing low- to moderate-intensity aerobic exercise sessions (lasting 30–40 min) such as yoga or aerobics for more than 20 weeks (2 times per week) can help to increase the mental energy level of PTSD patients and eliminate the psychological effects of PTSD-induced depression and depressive tendencies (12, 16). Empirical studies further support this, showing a 19% improvement in Negative Cognitions and Mood, a 21% enhancement in Re-experiencing, and a 17% reduction in Depressive Symptoms among exercise participants, thus contributing significantly to the mitigation of PTSD symptoms (20).

Overall, the primary mechanism by which exercise modulates SWB to ameliorate PTSD symptoms is by increasing military personnel’s positive emotions through exercise. This process effectively neutralises their negative emotions, gradually leading to emotional equilibrium and symptom relief in veterans.

#### 3.1.2 Exercise modulates PWB to improve PTSD symptoms

The concept of PWB differs from SWB by aligning with the self-deterministic philosophical tradition, which views happiness in terms of personal flourishing and the fulfilment of human potential (21). Mental health is closely related to human growth and development. Exercise affects veterans’ PWB by influencing dimensions such as determination and inner strength, as well as identity and self-acceptance.

In their study on military personnel scaling Mount Kilimanjaro, Burke et al. (22) revealed that mountaineering fostered determination and inner strength among veteran patients by presenting them with a significant goal to pursue. The challenging mountaineering environment and its physical rigours resonated deeply with their military backgrounds, inspiring a revival of purpose and direction in their lives. Achieving the exercise goals also contributed to PWB, giving the veterans a new sense of confidence and gradually alleviating the feelings of powerlessness associated with PTSD (22).

Exercise serves as a catalyst for promoting self-acceptance and cultivating new identities among veterans. It enables veterans to distance themselves from negative associations linked to the ‘mental illness’ label, fostering positive identities grounded in actions and accomplishments derived from their exercise narratives (13). For instance, despite the traumatic nature of mountaineering for these service members, it facilitated a reconnection with their wounded veteran identity, guiding a reflection on their intended path (22).

In general, participation in sport helps military personnel to find a balance between the feelings of powerlessness caused by traumatic events and the fulfilment that comes from participating in sport. This balance contributes to a renewed sense of accomplishment and self-confidence. This balance therefore promotes the mental health of veterans and helps to reduce and alleviate their PTSD symptoms.

### 3.2 Physiological mechanisms of exercise interventions for PTSD in veterans

Empirical studies indicate that the experience of PTSD affects an individual’s neurobiological functioning. Therefore, improving physiological factors through exercise can potentially mitigate physiological damage caused by PTSD, offering relief or potential treatment for veteran patients (23). Literature reviews highlight that exercise interventions primarily impact the physiological functions of brain structures, the neuroendocrine system—such as the Hypothalamic Pituitary Axis (HPA) and Neuropeptide—and the immune system among veteran patients. Below, I’ll outline the primary mechanisms underlying exercise intervention.

#### 3.2.1 Exercise modulates brain structure to improve PTSD symptoms

In terms of brain structure, the onset and severity of PTSD are associated with structural irregularities in both the grey matter and white matter of specific brain regions. Grey matter changes, including alterations in the volume of the hippocampus and anterior cingulate, or reductions in the volume or thickness of cortical brain tissues such as the praecuneus and insula, contribute to movement and memory dysfunctions in military individuals affected by PTSD (24, 25). Additionally, PTSD is linked to white matter abnormalities in the cingulum bundle, a white matter tract connecting the anterior medial prefrontal cortex to the medial temporal lobe, as well as in the superior longitudinal fasciculus, which connects the frontal and parietal lobes (26).

In response, academic theories built on the hypothesis that exercise can mitigate structural brain abnormalities in PTSD suggest that exercise-induced alterations in brain structure could alleviate PTSD symptoms (24). For instance, Colcombe et al. (14) conducted a study on aerobic training, revealing that aerobic exercise, conducted at 70% of heart rate reserve, led to an increase in the volume of prefrontal brain regions, notably the anterior cingulate gyrus, compared to a stretching control group. In addition, a positive correlation has been found between the level of motor ability and the volume of grey matter in the cingulate gyrus (both anterior and posterior cingulate gyrus), the left superior frontal gyrus, and the medial cortex of the left parietal lobe by using the cycle ergometer test (27). Supporting the idea that the physiological structure of the brain can be improved with the help of exercise training.

In addition, cardiorespiratory fitness testing using the Woodway Barimill treadmill has shown that aerobic exercise programmes can increase hippocampal brain volume by approximately 2%, mainly due to exercise-induced neurogenesis in the dentate gyrus subregion of the hippocampus (24). Consequently, as per the mechanistic theory positing that PTSD results in reduced hippocampal volume, exercise demonstrates potential efficacy in ameliorating the adverse effects of PTSD by fostering an increase in hippocampal volume (24).

#### 3.2.2 Exercise modulates the neuroendocrine system to improve PTSD symptoms

Veterans experiencing PTSD often display disruptions in their neuroendocrine system, leading to comorbidities like depression and anxiety. Empirical studies have indicated that dysfunctions within the neuroendocrine system play a crucial role in triggering depression (28). The Hypothalamic Pituitary Axis (HPA) is a fundamental mechanism affected in the high-stress conditions of PTSD. Here, the hypothalamus releases corticotrophin-releasing factor (CRF), stimulating the pituitary gland to release adrenocorticotropin hormone (ACTH), eventually prompting the release of cortisol. Dysregulation in this process is associated with psychiatric disorders like depression and anxiety (11). Moreover, a negative feedback mechanism exists within this process, regulating the body’s cortisol levels. However, in PTSD-affected individuals, this feedback system often malfunctions, hindering the body’s ability to self-regulate (28).

Exercise plays a dual role: it stimulates cortisol production and helps regulate the dysfunctional HPA system. By addressing the disruption of the HPA system caused by PTSD, exercise effectively alleviates depression caused by PTSD symptoms and reduces their impact (28). In essence, normalisation of the HPA axis and the release of hormones such as cortisol are closely linked to improvements in PTSD symptoms and represent a potential therapeutic target for the treatment of PTSD (29).

Several studies have shown that vigorous and consistent aerobic exercise has the potential to positively influence the HPA, leading to the normalisation and reduction of PTSD symptoms. For example, Galbo (30) observed that both vigorous and moderate-intensity exercise induced the secretion of ACTH and stimulated the release of cortisol. This process helps to regulate or ameliorate the dysfunction within the stress-related HPA, alleviating the negative feedback regulation of cortisol and ultimately alleviating PTSD symptoms (31). In addition, the release of hormones such as endorphins and the production of nerve growth factors such as brain-derived neurotrophic factor (BDNF) have been associated with improvements in depressed mood. It’s plausible that these hormones could also contribute positively to the treatment of PTSD (32).

However, the precise extent to which exercise influences hormone secretion and the expression of associated nerve growth factors to normalise HPA function for the treatment or alleviation of PTSD symptoms remains unclear. This aspect warrants further investigation in future studies.

Certainly, endogenous neuropeptides, such as neuropeptide Y (NPY), have shown positive effects in the treatment of PTSD. NPY, known for its anti-stress, pro-reward and anti-nociceptive properties, shows a significant negative correlation with PTSD symptoms (33). Research suggests that intense exercise, with an average maximal oxygen uptake of over 70%, triggers the release of NPY in humans, increasing its levels in the body. For example, Rämson et al. (34) found that high-intensity exercise, such as rowing training, over a 4-week period significantly increased the ability to release NPY. This effect of exercise-induced NPY elevation has been validated in veteran patients, strengthening the link between exercise and NPY-mediated effects on PTSD (33). Furthermore, the hypothesis linking vigorous exercise to increased NPY release appears to be linked to mechanisms that influence self-efficacy and intrinsic motivation (35). It’s clear that symptom improvement in veterans through exercise involves intertwined psychological and physiological factors that work synergistically, rather than independently.

#### 3.2.3 Exercise modulates immune system to improve PTSD symptoms

Absolutely, severe PTSD symptoms in veterans often coincide with heightened inflammation through a specific mechanism linking PTSD to pro-inflammatory cytokines like interleukin-1 beta (IL-1β), interleukin-6 (IL-6), interleukin-10 (IL-10), and tumor necrosis factor-alpha (TNF-alpha). These cytokines are associated with increased pro-inflammatory activity, significantly affecting physiological inflammation in veteran patients (36). As c-reactive protein levels rise in veterans with PTSD, anti-inflammatory markers such as interleukin-8 (IL-8) and interleukin-2 decrease, further intensifying inflammatory activity in these individuals. Hence, the primary mechanism of exercise intervention in PTSD involves impeding inflammation (15).

Certainly, stress responses associated with PTSD, like chronic high pressure, fear, and anxiety, trigger both central and peripheral immune cells to release pro-inflammatory cytokines such as IL-1β, IL-6, and others (15). For instance, in a social stress test, Bierhaus et al. (37) discovered that stress activates the inflammatory response in the neural periphery by enhancing nuclear factor κB (NF-κB) transcriptional activity. This activation results in increased circulating concentrations of IL-6, consequently heightening inflammation levels in PTSD patients.

Considering theoretical investigations into the immune system and PTSD mechanisms, scholars suggest the potential for exercise to boost the release of anti-inflammatory markers, thereby curbing inflammation and mitigating PTSD symptoms (15). In addition, aerobic exercise training for more than 4 h per week enhances immune system regulation by elevating circulating IL-10 and IL-1 receptor antagonists, suppressing the production of pro-inflammatory cytokines and interrupting pro-inflammatory signaling (38). This leads to a decrease in the frontal inflammatory response among individuals with PTSD (38).

Finally, studies indicate a notable negative link between pro-inflammatory cytokines like IL-1β, TNF-*α*, and non-rapid eye movement sleep, suggesting that higher cytokine concentrations correlate with poorer sleep quality in PTSD patients (39). Consequently, modulating the decrease in pro-inflammatory cytokines via exercise might enhance sleep quality and alleviate PTSD symptoms. While the positive impact of exercise on inflammation and its sleep-regulating potential appears promising for PTSD intervention, this notion warrants substantiation through further empirical research (11). In essence, exercise-induced activation of the neuroendocrine system holds promise for immune system modulation, potentially ameliorating inflammation levels in PTSD patients (15).

## 4 Conclusion

In conclusion, exercise interventions, especially as part of a comprehensive therapy, may be effective in reducing the stigma and discomfort that service members experience as a result of being treated for PTSD, as well as reducing the risk of suicide that they face after discharge from the military.

However, it is worth noting that although this study analysed the mechanisms of exercise interventions for the treatment of PTSD in veterans, there is a lack of more discussion about exercise co-interventions for the treatment of PTSD. For example, there is a lack of discussion about the negative effects of exercise interventions, such as injuries sustained during exercise, which may cause negative effects such as their stress response. This needs to be addressed in future studies. In addition, we found that there is a lack of higher quality randomised controlled trials in the existing trials to further explore the exercise dose (exercise programme, exercise duration, exercise intensity, etc.) required for a specific exercise intervention. For this reason, although this paper is not focused on exercise interventions, in order to present the programs covered in this paper more clearly and to inform subsequent research or intervention practice, this paper provides more detailed exercise programs, as detailed in Tables 1, 2.

**Table 1:** Summary of exercise types and exercise intervention programmes for outdoor exercise.

Type of sport (References)	Exercise intervention programme
Mountaineering (22)	Participants are arranged to summit the 5,895-metre mountain (Mount Kilimanjaro) in six stages over a nine-day period, with camping at five different altitudes along the way. The recommended altitude for the first stage is 2,650 m (Simba Camp); for the second stage, 3,450 m (2nd Caves Camp); for the third stage, 3,600 m (Kikelewa Cave); for the fourth stage, 4,330 m (Mawenzi Tarn Camp); Stage 5 with a recommended altitude of 4,700 m (stationed at Kibo Hut); Stage 6 for the final ascent
Surfing (47)	Arrangements are made for participants to start with 1 h of yoga to relax and stretch before embarking on a surfing programme of 3–4 h at a time for 6 weeks. Participants are free to take breaks of their choice during the surfing period. During this 6-week period, it is recommended to have surfing activities during the day and tea parties in the evenings to help participants rest and regroup.
Hiking (42)	Arrange for participants to go hiking 4 or 6 times a week for 12 weeks. Suggested exercises include a 3–5 min warm-up, keeping the length of the hikes within 1–1.5 h and 2–3 miles initially, and then gradually increasing the length of the hikes to 2–3 h and 5–6 miles. In addition, during the hike, breaks were recommended every 30 min.
Rafting (40)	Participants are scheduled to engage in outdoor ‘river running’ over a 4-day period, where participants are advised to wake up at dawn, eat breakfast, and dismantle their makeshift campsites each day. Rafting takes place during the day (morning and afternoon), lunch is disposed of ashore, campsites are set up in the evening, and a campfire is organised in the evening to allow participants to relax and rest. The duration and distance of the rafting is not required, just follow the natural flow of the river and find a suitable stopping point near lunch and dinner time.
Outdoor adventure (48)	Participants are arranged to participate in the wilderness ‘Outward Bound Experience’ over a period of 5 days. It is recommended that 1 day of preparation be used to complete the setting of research objectives, group exercises on cooperation and trust, and departure to the experience site; followed by 3 days of the wilderness ‘Outward Bound Experience’ programme; and 3 days of the final wilderness ‘Outward Bound Experience’ programme. This is followed by a 3-day wilderness ‘outreach training experience’ programme; and a return trip on the last day. It is suggested that the course should include a combination of standardised experiential tasks, including how to tie knots, rock climbing, hiking, camping and rafting.

**Table 2:** Summary of exercise types and exercise intervention programs for other exercises.

Type of sport (References)	Exercise intervention programme
Aerobic (12, 16)	Participants underwent a 40-week, twice-weekly aerobic exercise programme. Exercise sessions consisted of low- to moderate-intensity exercises accompanied by music, including a 5-min warm-up, 30 to 40 min of aerobic training (e.g., aerobics), about 10 min of muscular strength and endurance exercises (sit-ups, push-ups, or resistance exercises), and finally muscle relaxation through flexibility and stretching exercises.
Aerobic + resistance (2, 10, 20)	Schedule participants for 12–20 weeks of moderate to vigorous intensity aerobic and resistance training 3 times per week for 30–60 min in duration, of which more than 150 min per week should be exercised. It is recommended to arrange simple aerobic training or resistance or mixed training with the help of equipment such as treadmills, stationary bikes and dumbbells, depending on the different states of the participants.
Mixed exercise (17, 43)	Arranging 5 consecutive days of physical activities for the participants. It is recommended that the daytime participants take part in various adaptive sports (e.g., wheelchair basketball, wheelchair badminton, seated volleyball, archery, indoor bowling) and adventurous training (e.g., indoor rock-climbing, avalanches, skeet-shooting, canoeing), and that a tea party be held in the evening to conduct interviews and communication with the participants.

From the above outdoor exercise interventions, the following key points of outdoor exercise intervention settings can be summarised: firstly, the duration of outdoor exercise interventions can be short (ranging from 3 days to 12 weeks), but veterans need to be fully exposed to nature and live in natural environments (40); secondly, there is no limit to the intensity of outdoor exercise interventions and participants are asked to do their best and live within their means, for example, there is an emphasis on the absence of specific goals, such as the ‘going with the flow’ of rafting (40), where participants are programmed for up to a few hours but can stop at any time (47); thirdly, there are no clear restrictions on outdoor exercise sports as long as they are in contact with nature, such as outdoor skiing, camping, fishing, etc.; fourthly, if the goal of the sport is to be set, it needs to be set as a way of learning new knowledge and skills and diverting their attention, rather than adding a psychological burden of competition for them fifth, and most importantly, for military personnel, training in nature is the most ‘familiar’ way for them to train in nature. Fifth, and most importantly, for military personnel, training in nature is the most ‘familiar’ scenario for them, and this is also the specific scenario that may cause them to suffer from PTSD. Therefore, when implementing outdoor sports interventions for military personnel, it is necessary to understand the history of PTSD beforehand and, as far as possible, avoid ‘returning to the scene’ of an unfamiliar situation that may lead to deeper harm, ‘leading to deeper harm’ (5).

Similar to outdoor exercise interventions, exercise interventions for veteran patients through exercise programmes such as aerobic exercise, aerobic + resistance training, and mixed exercise require attention to develop a sense of security and confidence in the service member and awareness of tasks that may trigger distress (41). It is also important to provide clear information and explanations of the task so that, at a higher cognitive level, the service member is less likely to experience the distracting effects of exercise due to unnecessary contemplation (41). It is also important to note that although military personnel are more familiar with exercise training, it is still important to avoid injuries due to excessive exercise loads, so exercise prescriptions need to be set up with specific individuals in mind.

In summary, although this study primarily examined exercise interventions in a population of veteran patients, its implications extend greatly to active duty military personnel at risk for PTSD. This study has considerable value for the development of mental health programmes within the military. In addition to military personnel, a variety of frontline groups who have to deal with traumatic experiences, such as peacekeepers, firefighters, disaster survivors, health care workers, and residents of outbreak areas, could also benefit from exercise interventions targeting PTSD. Therefore, the results of this study are relevant to the design of interventions and treatments for PTSD in these different groups.
